# Genome-Wide Identification and Expression Analysis of *BraGLRs* Reveal Their Potential Roles in Abiotic Stress Tolerance and Sexual Reproduction

**DOI:** 10.3390/cells11233729

**Published:** 2022-11-22

**Authors:** Liu Yang, Yumei Zhao, Xiaoyu Wu, Yang Zhang, Yehan Fu, Qiaohong Duan, Wei Ma, Jiabao Huang

**Affiliations:** 1 State Key Laboratory of Crop Biology, Shandong Agricultural University, Tai’an 271000, China; 2 College of Horticulture Science and Engineering, Shandong Agricultural University, Tai’an 271000, China; 3 College of Horticulture, Hebei Agricultural University, Bao’ding 071000, China; 4 State Key Laboratory of North China Crop Improvement and Regulation, Bao’ding 071000, China

**Keywords:** *Brassica rapa*, *GLR* gene family, expression profile, abiotic stress, sexual reproduction

## Abstract

Glutamate receptors (GLRs) are involved in multiple functions during the plant life cycle through affecting the Ca^2+^ concentration. However, GLRs in Brassica species have not yet been reported. In this study, 16 glutamate receptor-like channels (GLR) belonged to two groups were identified in the *Brassica rapa* (*B. rapa*) genome by bioinformatic analysis. Most members contain domains of ANF_receptor, Peripla_BP_6, Lig_chan, SBP_bac_3, and Lig_chan_Glu_bd that are closely related to glutamate receptor channels. This gene family contains many elements associated with drought stress, low temperature stress, methyl jasmonate (MeJA), salicylic acid (SA), and other stress resistance. Gene expression profiles showed that *BraGLR* genes were expressed in roots, stems, leaves, flowers, and siliques. *BraGLR5* expression was elevated after drought stress in drought-sensitive plants. *BraGLR1*, *BraGLR8*, and *BraGLR11* expression were significantly upregulated after salt stress. *BraGLR3* expression is higher in the female sterile-line mutants than in the wild type. The expression levels of *BraGLR6*, *BraGLR9*, *BraGLR12*, and *BraGLR13* were significantly higher in the male sterile-line mutants than in the wild type. The expression of most *BraGLRs* increased after self-pollination, with *BraGLR9* exhibiting the greatest increase. These results suggest that *BraGLRs* play an important role in abiotic stress tolerance and sexual reproduction.

## 1. Introduction

Glutamate receptors were originally discovered in animals and include ion channel glutamate receptors (iGluRs) and metabolic glutamate receptors (mGluRs). Lam et al. [1] identified ionotropic GluRs in plants: isolated *GLR1* and *GLR2* from *Arabidopsis thaliana* (*A. thaliana*). Subsequently, 20 members of the glutamate receptor-like gene family were identified in *A. thaliana* [2]. As a Ca^2+^ channel protein, GLRs are ligand-gated ion channels [3], also known as ion receptors. These transmembrane ion channel proteins can respond to ligand signals, opening ion channels to mediate cation entry into the cell interior and affecting intracellular ion concentration; thus, they are involved in many biological processes [4].

GLRs participate in resistance to biotic and abiotic stresses. *AtGLR1.2* and *AtGLR1.3* promote the downstream CBF/DREB1 cold response pathway by regulating endogenous jasmonic acid (JA) levels, thereby enhancing plant cold tolerance [5]. Glutamate (GLU) can induce Ca^2+^ inward flow by activating GLRs, and [Ca^2+^]_cyt_ can be decoded by calmodulins and further enhance plant heat tolerance [6]. *AtGLR3.7* is involved in the salt stress response in *A. thaliana* by influencing the calcium signaling pathway [7]. The glutamate receptor of small radish (RsGluR) is a glutamate-gated Ca^2+^ channel located on the plasma membrane of higher plants and acts in defense against pathogenic bacterial infection by triggering JA biosynthesis [8]. Cold, touch, and glutamate all activate the transcription of *AtGLR3.4* in a calcium-dependent pathway [9]. As calcium homeostasis-related proteins, GLRs are involved in mediating glutathione-triggered cytoplasmic calcium transients and transcriptional changes, with a role in the innate immune response [10].

GLRs are also involved in sexual reproduction. GLRs promote Ca^2+^ influx through the plasma membrane and regulate the apical cytosolic Ca^2+^ concentration ([Ca^2+^]_cyt_) gradient, thereby influencing Ca^2+^ inward flow-dependent pollen tube growth and function [11,12]. Iwano et al. [13] found that the [Ca^2+^]_cyt_ in stigma papilla cells increases sharply after self-pollination in self-incompatible *A. thaliana* and can be effectively blocked by inhibitors of GLR. Additionally, in glutamate receptor-like mutants, the increase in Ca^2+^ caused by self-incompatibility is reduced, and GLRs can mediate Ca^2+^ influx, suggesting that GLR-mediated Ca^2+^ influx in stigma papilla cells is a key factor leading to plant rejection of its own pollen. Ca^2+^ was also reported to be involved in the self-incompatibility and self-compatibility response of *Brassica napus* [14].

GLRs are also involved in light signal transduction [1], C and N metabolism, abscisic acid (ABA) biosynthesis, signal transduction [15,16], Ca^2+^ influx [17], stomatal movement [18], cell division and single cell survival [19], calcium permeation [3], and cellular homeostasis [17].

*B. rapa* is a Brassica vegetable crop and a typical sporophyte self-incompatibility plant that is widely cultivated and consumed. During cultivation, abiotic stresses, such as drought and salt, can severely affect yield and quality. Although GLRs play key roles in modulating these stresses in other plants, the *BraGLRs* were not previously identified. In this study, we identified and analyzed the expression profiles of *BraGLR* genes after abiotic stress treatment and pollination response. The results of this work provide a basis for subsequent in-depth studies on the function of the *BraGLR* genes.

## 2. Materials and Methods

### 2.1. Identification, Physicochemical Characterization, and Subcellular Localization of BraGLRs

*A. thaliana* genomic data were downloaded from TAIR (https://www.arabidopsis.org/ accessed on 20 July 2022) and *B. rapa* genomic data were downloaded from BRAD (http://brassicadb.cn/ accessed on 20 July 2022) to construct a local BLAST database. Preliminary screening of the GLR gene family members in *B. rapa* was performed using the sequences of 20 known GLR gene family members in *A. thaliana* (*AtGLRs*) by bidirectional BLAST (E-value < 1 × 10^−10^, Identity > 40%). The conservation of domains of the selected members was analyzed by NCBI’s Conserved Domain Database (https://www.ncbi.nlm.nih.gov/cdd accessed on 20 July 2022) [20], and the domains were verified by Pfam (E-value < 1.0) (http://pfam.xfam.org/ accessed on 20 July 2022) [21]. Members that did not contain typical domains were eliminated to finally identify the members of the *BraGLRs*. Analysis of physicochemical properties such as molecular weight (MW), isoelectric point (PI), and amino acid length of the finalized set of *BraGLR* genes was performed using Expasy (https://web.expasy.org/protparam/ accessed on 20 July 2022). Subcellular localization prediction was conducted using the WOLF PSORT website (https://www.genscript.com/wolf-psort.html accessed on 20 July 2022) [22].

### 2.2. Construction of Phylogenetic Tree and Synteny Analysis

The MLE (Maximum Likelihood Estimate) phylogenetic tree of *BraGLRs* and *AtGLRs* and the phylogenetic tree of *BraGLRs* were constructed using MEGA-X (bootstrap value set at 1000) [23]. The phylogenetic tree was modified for clearer presentation using iTOL (https://itol.embl.de/ accessed on 20 July 2022) [24].

Synteny was analyzed with the Advanced Circles and Table Row Extract or Filter programs of TBtools [25].

### 2.3. Gene Structure and Protein Domain Analysis

The functional structure domain information of *BraGLRs* was obtained using Pfam (http://pfam.xfam.org/ accessed on 20 July 2022) and visualized using Visualize Domain Pattern (from Pfam Search) of TBtools [21]. The structure of *BraGLRs* was visualized using the Visualize Gene Structure (Basic) program of TBtools. The online program MEME 5.4.1 (https://meme-suite.org/meme/tools/meme accessed on 20 July 2022) was used to analyze the conserved motifs of the *BraGLRs* (predicted number of motifs, 10; other parameters were set to the default values) [26].

### 2.4. Analysis of Promoter Cis-Regulatory Elements, GO Enrichment Analysis, and Transcriptome Data Expression

The sequences of the 2 kb upstream of the start codon of *BraGLRs* were extracted in Ensemble Plants (http://plants.ensembl.org/index.html accessed on 20 July 2022). The cis-acting element characteristics were predicted and analyzed using the Search for CARE function of PlantCARE (http://bioinformatics.psb.ugent.be/webtools/plantcare/html/, accessed on 20 July 2022) with the default parameters [27].

GO Enrichment Analysis can classify and analyze GLR members according to their functional similarity. The *BraGLR* genes’ IDs were uploaded to the online DAVID database (https://david.ncifcrf.gov/, accessed on 20 October 2022). Then, the GO annotation data were handled and graphical demonstration was given by using bioinformatics (http://www.bioinformatics.com.cn/, accessed on 20 October 2022).

Transcriptome data were obtained to analyze tissue-specific expression of *B. rapa*, female sterile mutants, male sterile mutants, drought-tolerant, and drought-sensitive plants in the Brassicaceae Database (http://brassicadb.cn/ accessed on 20 July 2022) using the HeatMap plug-in for TBtools and Excel 2016 for graphing.

### 2.5. Total RNA Extraction and qRT-PCR

*B. rapa* seedlings were treated in Hoagland nutrient solution prepared with 150 mmol L^−1^ NaCl solution for 2, 4, 6, and 12 h, and normal hydroponic seedlings were used as the control, for a total of five treatments. There were three biological replicates of each treatment. The stigmas of *B. rapa* were unpollinated (UP), self-pollinated (SI), and cross-pollinated (CP), for 10 min, with three treatments.

Total RNA was extracted using the SteadyPure Universal RNA Extraction Kit (Accuratel Biology, Hunan, China), followed by a reverse transcription reaction with a HiScript^®^ II QRT SuperMix for qPCR Sample Kit (Vazyme, Nanjing, China) to obtain cDNA. The qRT-PCR primer sequences (Table S1) were designed using the qPrimerDB-qPCR Primer Database (https://biodb.swu.edu.cn/qprimerdb/ accessed on 20 July 2022) [28] and synthesized by Qingdao PrimeTech Zixi Biotechnology Co. Ltd. The qRT-PCR reactions were performed on a QuantStudio 3 system (Applied Biosystems, Massachusetts, USA) with ChamQ SYBR qPCR Master Mix (Vazyme, Nanjing, China), with *BraActin2* used as the internal control. Reactions were performed with three technical repetitions. The data were analyzed using the 2^−∆∆CT^ method [29], and the data were analyzed and graphed using Excel 2016.

### 2.6. Analysis of Protein Secondary Structure, Tertiary Structure, and Protein Interaction Network

The protein secondary structure of BraGLRs was analyzed by PRABI (https://npsa-prabi.ibcp.fr/cgi-bin/npsa_automat.pl?page=npsa_sopma.html accessed on 20 July 2022), and the results were analyzed and plotted using Excel 2016.

Predictive analysis of the tertiary structure of BraGLR proteins was performed through the SWISS-MODEL (https://swissmodel.expasy.org/ accessed on 20 July 2022) [30], and the tertiary structure images of each member were compiled by Adobe Photoshop CC 2019 for this study.

For protein interaction network analysis, the IDs of BraGLRs were entered in the Protein Interaction Network Prediction website (https://cn.string-db.org/cgi/input?sessionId=bnElPBJqHKQ5&input_page_active_form=single_identifier accessed on 20 July 2022) with selection of *B. rapa* as the organism to obtain the protein network interactions map (minimum required interaction score = 0.150, default settings were used for the other parameters) [31].

## 3. Results

### 3.1. Identification, Physicochemical Characterization, and Subcellular Localization of BraGLRs

A sequence comparison analysis was performed by HMMER and local BLAST, and a total of 20 *BraGLR* members were obtained by homology matching with the *B. rapa* genome database. The sequences were analyzed using NCBI’s Batch CD-Search and Pfam; four members not containing typical domains were found and removed, resulting in a total of 16 *BraGLR* members. The *BraGLR* members are named *BraGLR1*-*BraGLR16* in order, according to their physical position on the chromosome (from left to right, top to bottom). The analysis of the basic physicochemical properties of all *BraGLRs* revealed amino acid lengths ranging from 593 aa (*BraGLR14*) to 1856 aa (*BraGLR3*), PI values ranging from 6.10 (*BraGLR2*) to 8.77 (*BraGLR14*), and molecular weights ranging from 66681.35 (*BraGLR14*) to 207961.61 (*BraGLR3*). Next, the subcellular localizations of BraGLRs were predicted, and the results indicate that all proteins localized to the plasma membrane (plas), as well as BraGLR7, BraGLR8, BraGLR14, and BraGLR16 proteins, were predicted to be localized to the nuclear matrix (nucl), chloroplast (chlo), endoplasmic reticulum (E.R.), and cytosol (cyto), respectively (Table 1). The results suggest multiple roles for the members of this gene family.

### 3.2. Phylogenetic Relationships and Synteny Analysis of BraGLRs

**Figure 1:**
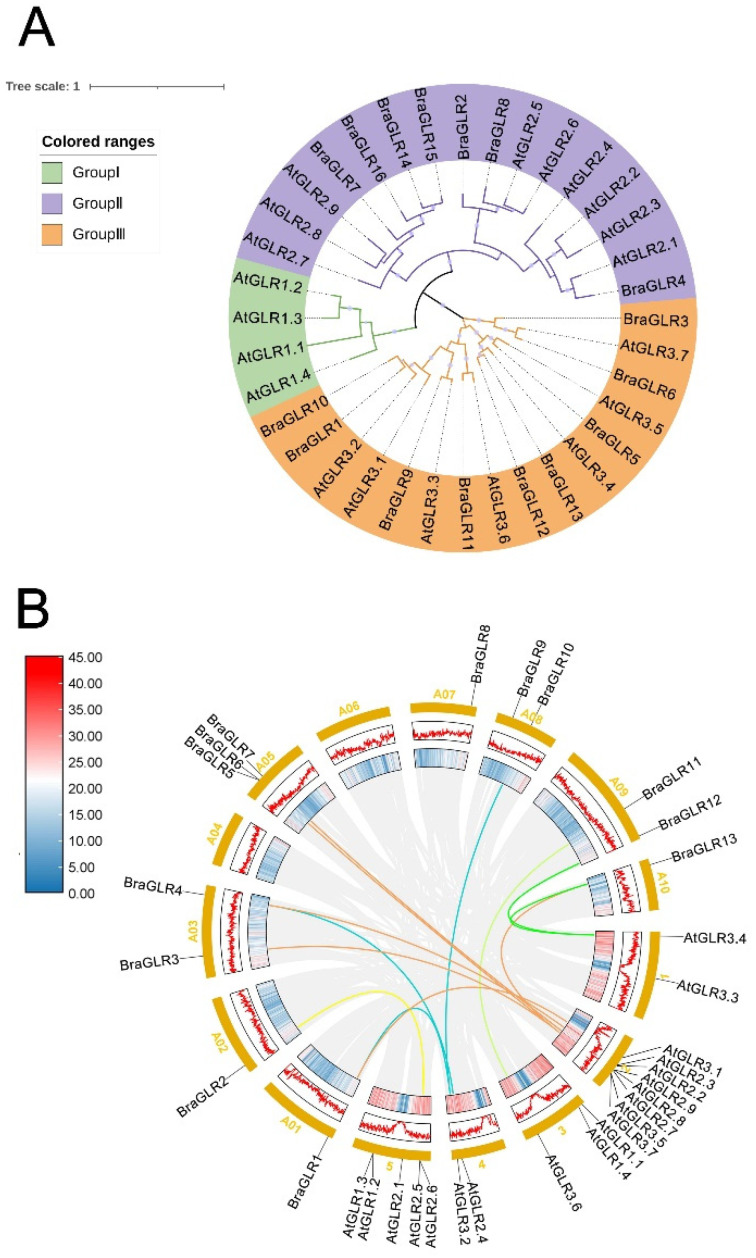
Phylogenetic relationships and synteny analysis of GLR genes between *B. rapa* and *A. thaliana*. (**A**) Phylogenetic relationships. (**B**) Synteny analysis. The curves of green, orange, yellow green, blue, and yellow link the *GLR* genes on the Chr1, Chr2, Chr3, Chr4, and Chr5 chromosomes of *A. thaliana* and orthologous genes in *B. rapa*, respectively.

To understand the kinship and evolutionary characteristics among *GLR* family members, evolutionary trees of the *GLR* gene families of *B. rapa* and *A. thaliana* were constructed based on sequence similarity (Figure 1A). *AtGLRs* were divided into three groups (Group I, Group II, and Group III). The analysis of the evolutionary trees results did not screen for *BraGLRs* with high homology to *AtGLR* Group I; therefore, the *BraGLR* members were classified into two groups (Group II and Group III). The *BraGLR* members corresponded to two groups in *AtGLRs*: Group II had seven members and Group III had nine members.

The 16 *BraGLR* genes were mapped to 8 of the 10 chromosomes of *B. rapa* (Figure 1B). Chromosomes 4 and 6 had no *BraGLR* genes, *BraGLR14*, *BraGLR15*, and *BraGLR16*, were distributed on the chromosome scaffold, there was one *BraGLR* gene on each of chromosomes 1, 2, 7, and 10, and two *BraGLR* genes on each of chromosomes 3, 8, and 9. Three genes mapped to chromosome 5, the chromosome with the largest number of *BraGLR* members, and there were two separate pairs of tandem repeats in this gene family: *BraGLR1* and *BraGLR10*, and *BraGLR12* and *BraGLR13*. To better understand the evolutionary relationships and phylogenetic mechanisms among the *BraGLRs*, a synteny analysis map of the GLRs was constructed using the genes of *A. thaliana*, a model plant belonging to the Brassicaceae, as reference (Figure 1B). Most *BraGLR* genes were found to be covalently related to the *AtGLR* genes, suggesting that the genes may have similar biological functions and providing a direction to explore the functions of *BraGLRs*.

### 3.3. Gene Structure and Protein Domain Analysis of BraGLRs

**Figure 2:**
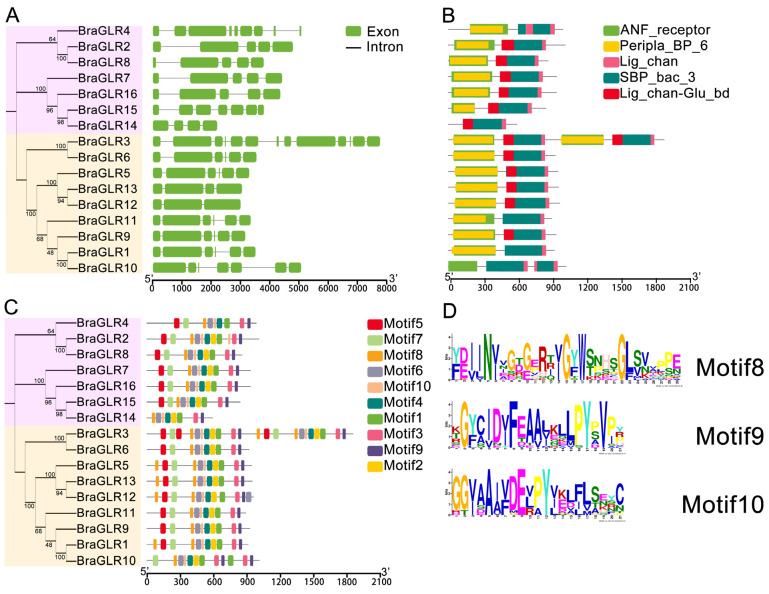
Structure and conserved domain analysis of GLRs in *B. rapa*. (**A**) Gene structure of the *GLR* genes in *B. rapa*. The exons and introns are represented by green boxes and black lines, respectively. (**B**) Analysis of the functional structural domain of *GLR* genes in *B. rapa*. (**C**) Analysis of the conserved structural domain of *GLR* genes in *B. rapa*. (**D**) Motif sequence. See Figure S1 for detailed motif information.

To analyze the similarities and differences of *GLR* genes at the nucleic acid and protein levels and to speculate on the structural, functional, and evolutionary relationships of these genes, the domains of *BraGLRs* were identified by Pfam (Figure 2B). The results show five functional domains commonly present in this gene family: ANF_receptor, Peripla_BP_6, Lig_chan, SBP_bac_3, and Lig_chan_Glu_bd. All members have Lig_chan and SBP_bac_3 domains, and all members except *BraGLR14* have the ANF_receptor and Peripla_BP_6 domains. The presence of these conserved domains indicates that the family has ligand family traits, facilitating the analysis of these genes.

The gene structure analysis showed (Figure 2A) that *BraGLRs* contain exons and introns, with the number of exons varying from 3 to 13. The number and distribution of exons in Group II are not regular, while the number and distribution of exons of members in Group III are relatively similar. Differences in gene structure can affect function, suggesting that Group III members may be functionally similar and can be further analyzed for function.

The conservative structural domain analysis shows (Figure 2C) that all *BraGLR* members contain Motif 1, Motif 3, Motif 4, Motif 6, Motif 8, Motif 9, and Motif 10, indicating that these are conserved motifs of the *BraGLRs*. There was a similar distribution of motifs in the same group, especially in Group III, where the number and position distribution of most members’ motifs are more consistent and highly conserved. The *BraGLRs*’ motif logo (Figure 2D and Figure S1) reveals that all motifs contain more conserved amino acids, indicating that these genes are highly conserved.

### 3.4. Analysis of Promoter Cis-Regulatory Elements of BraGLRs

**Figure 3:**
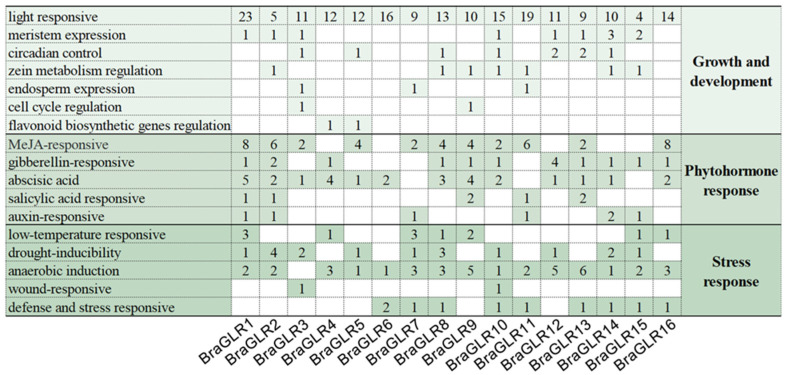
Analysis of *cis* elements in promoter of *GLR* genes in *B. rapa*.

To investigate the spatial and temporal characteristics of GLR gene expression in plant growth and defense systems, the 2 kb nucleic acid sequences upstream of the start codon of *BraGLRs* were analyzed by using PlantCARE (Figure 3). These upstream sequences contain a large number of light-responsive elements, suggesting that the expression of these genes may be regulated by light signals. Except for *BraGLR3*, all members contain anaerobic induction regulatory elements. About 80% of the members contain ABA elements, 70% contain MeJA-responsive elements, and about 60% contain drought-inducibility responsive elements. These results suggest that members of this gene family contribute to plant resistance to stresses [32].

### 3.5. Functional Annotation Analysis of BraGLRs

**Figure 4:**
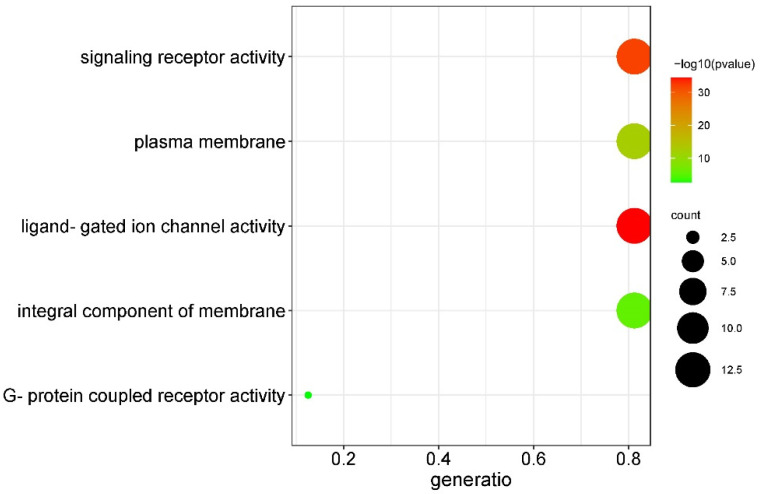
GO enrichment analysis of BraGLRs. The size of the dot bubble represents the number of genes on that GO term.

To further explore the function of the BraGLRs, a functional analysis using GO annotation and enrichment terms such as molecular function (MF), cellular component (CC) and biological process (BP) was investigated. However, the family was not enriched in BP (Figure 4). The GO-MF (Molecular Function) enrichment results detected three enriched terms, namely, ligand-gated ion channel activity (GO:0015276), signaling receptor activity (GO:0038023), and G protein-coupled receptor activity (GO:0004930). The GO-CC (cellular component) enrichment results detect two enriched terms, including the plasma membrane (GO:0005886) and an integral component of the membrane (GO:0016021). BraGLRs had 13 genes enriched on both ligand-gated ion channel activity and signaling receptor activity terms, with small *p*-values and high confidence, two genes enriched on G protein-coupled receptor activity term, 13 genes enriched in the plasma membrane term, and 13 genes enriched in the integral component of the membrane term.

### 3.6. Gene Expression Analysis of the BraGLRs

#### 3.6.1. Analysis of Tissue-Specific Expression of BraGLRs

**Figure 5:**
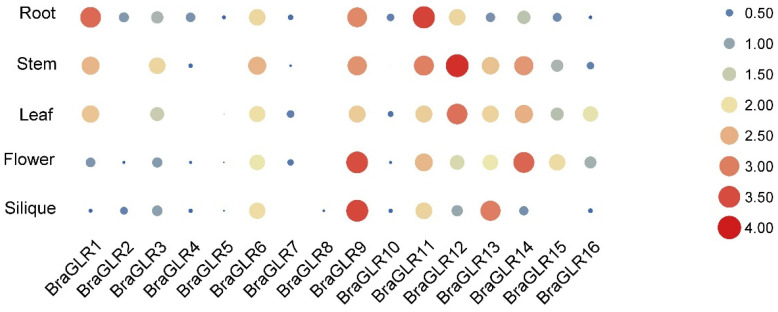
Transcript level analysis of *GLR* genes in different tissues of *B. rapa*. Larger area and darker color indicate higher expression. All values underwent logarithmic transformation.

To explore the expression of *BraGLRs* in different parts of *B. rapa* plants, tissue-specific transcriptomic data were obtained from the Brassicaceae Database. The analysis of transcriptomic data of *BraGLRs* extracted from five different tissues (root, stem, leaf, flower, and silique) (Figure 5) showed that *BraGLR9* was more highly expressed in flowers and siliques, *BraGLR11* is highly expressed in roots, and *BraGLR12* is highly expressed in stems. These clear tissue expression differences among *BraGLR* genes suggest different roles in different stages of plant development.

#### 3.6.2. Analysis of Abiotic Stress Transcript Levels of BraGLRs

**Figure 6:**
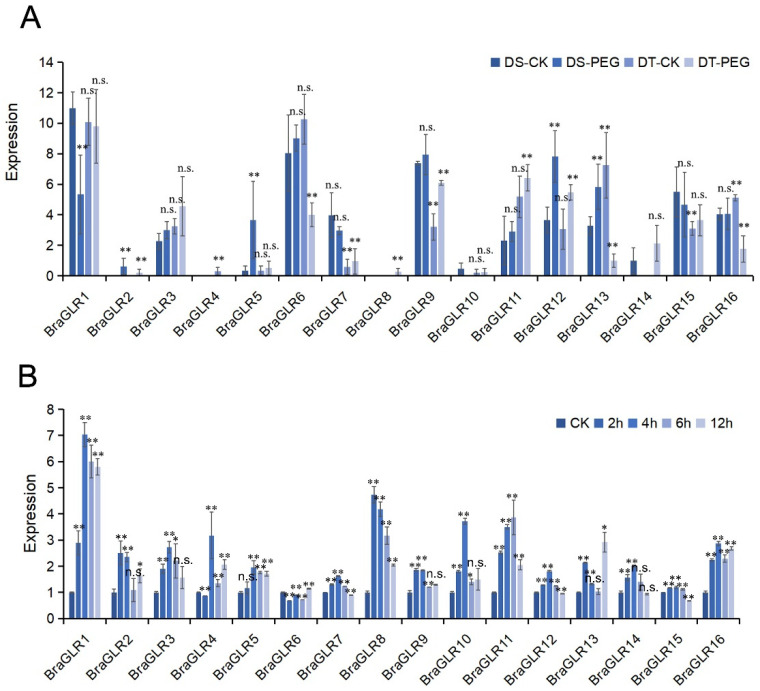
Analysis of abiotic stress transcript levels of *GLR* genes in *B. rapa*. (**A**) Analysis of drought stress transcriptome data of *GLR* genes in *B. rapa*. PEG treatment for drought-tolerant and drought-sensitive plants, denoted by DT-PEG and DS-PEG, respectively. (**B**) Analysis of salt stress relative expression of *GLR* genes in *B. rapa* by qRT-PCR. Eleven-day-old seedlings were treated with 150 mmol L^−1^ NaCl, followed by sampling at 0 (CK), 2, 4, 6, and 12 h. (n.s., no significance, * *p* < 0.05, ** *p* < 0.01, *t* test).

The analysis of transcriptome sequencing data for drought-sensitive (DS) and drought-tolerant (DT) *B. rapa* revealed an elevated expression of *BraGLR5* after drought stress of drought-sensitive plants (Figure 6A). According to the promoter element analysis (presented above), this gene contains a drought inducibility element, consistent with its significant response in DS plants. After drought stress treatment, DT plants had lower expression levels of *BraGLR9* and *BraGLR12* than DS plants. These two genes both have MeJA-responsive elements, and *BraGLR9* also has salicylic acid-responsive elements, consistent with the upregulation of these genes related to jasmonate and salicylate metabolism in response to drought stress [33].

The relative expression levels of *BraGLRs* were generally upregulated after salt stress. *BraGLR1*, *BraGLR8*, and *BraGLR11* were upregulated significantly, 5–7-fold compared to CK (Figure 6B). The analysis of promoter elements revealed that these three genes contain many MeJA-responsive elements. There was no obvious upregulation of *BraGLR6* and *BraGLR15*, and these genes lack MeJA-responsive elements. Numerous data show that methyl jasmonate can effectively mitigate the damage of salt stress on plants [34], consistent with the observed changes in expression detected by qRT-PCR.

#### 3.6.3. Sexual Reproduction-Related Expression Profiling of BraGLRs

**Figure 7:**
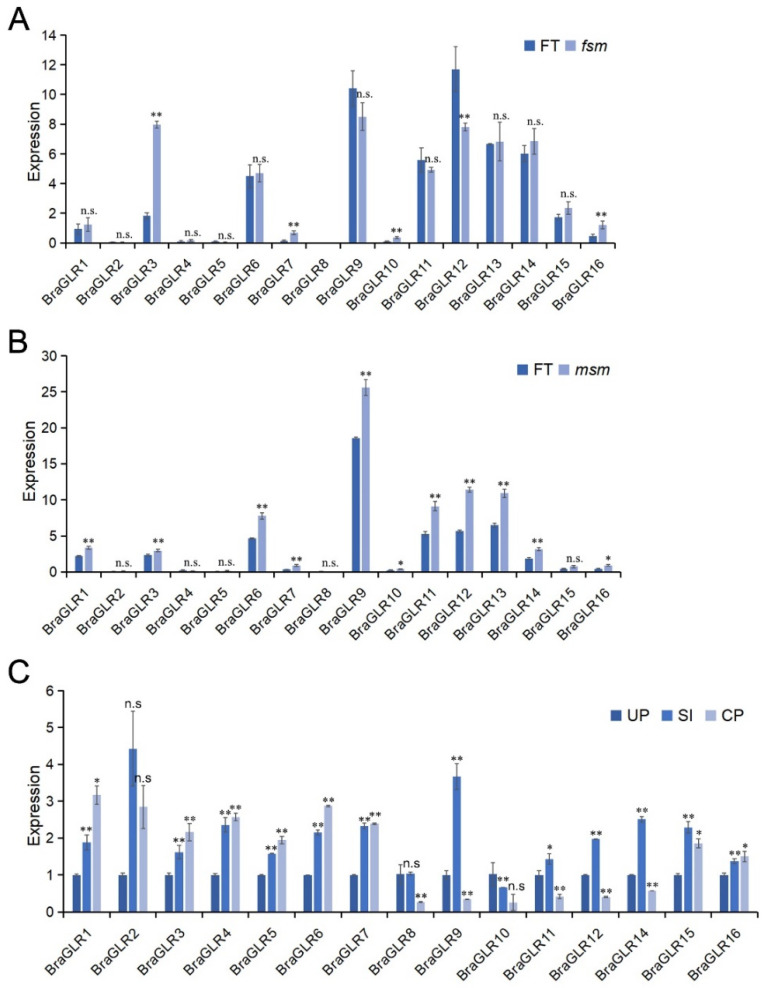
Analysis of sexual reproduction-related expression profiles of the *GLR* genes in *B. rapa*. (**A**) Analysis of female sterile mutant (*fsm*) and wild-type (FT) transcriptome data of *GLR* genes in *B. rapa*. (**B**) Analysis of male sterile mutant (*msm*) and wild-type (FT) transcriptome data of *GLR* genes in *B. rapa*. (**C**) Differential expression analysis of UP, SI, and CP. The stigmas of unpollinated, self-pollinated, and cross-pollinated *B. rapa* are denoted by UP, SI, and CP, respectively. (n.s., no significance, * *p* < 0.05, ** *p* < 0.01, *t* test).

The reproductive organs of plants are highly complex, and the study of female sterile mutants (*fsm*) could provide additional insights into the mechanisms regulating flower development. An *fsm* was obtained from *B. rapa* DH line ‘FT’ using a combination of isolated microspore culture and ethyl methanesulfonate (EMS) mutagenesis, with female sterility resulting from abnormal ovule development [35]. The analysis of transcriptome data from ‘FT’ and *fsm* (Figure 7A) revealed an elevated expression of *BraGLR3* in the female sterile-line mutant, suggesting that this gene may regulate pistil growth and development in *B. rapa*.

Among vegetables of the Brassica genus, F1 hybrids have the advantages of high yield, good quality, and resistance to adversity stress [36]. Male sterile lines were developed with high seed production efficiency and purity, and male sterile transcriptome data [37] were analyzed for changes in the expression of *BraGLRs* by comparing *B. rapa* male sterile mutants (*msm*) and the wild type (FT) (Figure 7B). The expression levels of *BraGLR6*, *BraGLR9*, *BraGLR12*, and *BraGLR13* were significantly elevated in *msm*, indicating that these genes help regulate the process of stamen growth and development in *B. rapa*.

The expression levels of the *BraGLRs* were measured under different pollination conditions using qRT-PCR, with expression levels in unpollinated plants as a control (Figure 7C). The results show a relatively elevated expression of most *BraGLRs* under self-pollination conditions, and a reduced expression of *BraGLR8*, *BraGLR9*, *BraGLR10*, *BraGLR11*, *BraGLR12*, *BraGLR14*, and *BraGLR15* after cross-pollination. The expression of *BraGLR9* was significantly elevated after the self-incompatibility response, indicating that these genes act in sexual reproduction.

### 3.7. Analysis of BraGLRs’ Protein Secondary Structure and Tertiary Structure

**Figure 8:**
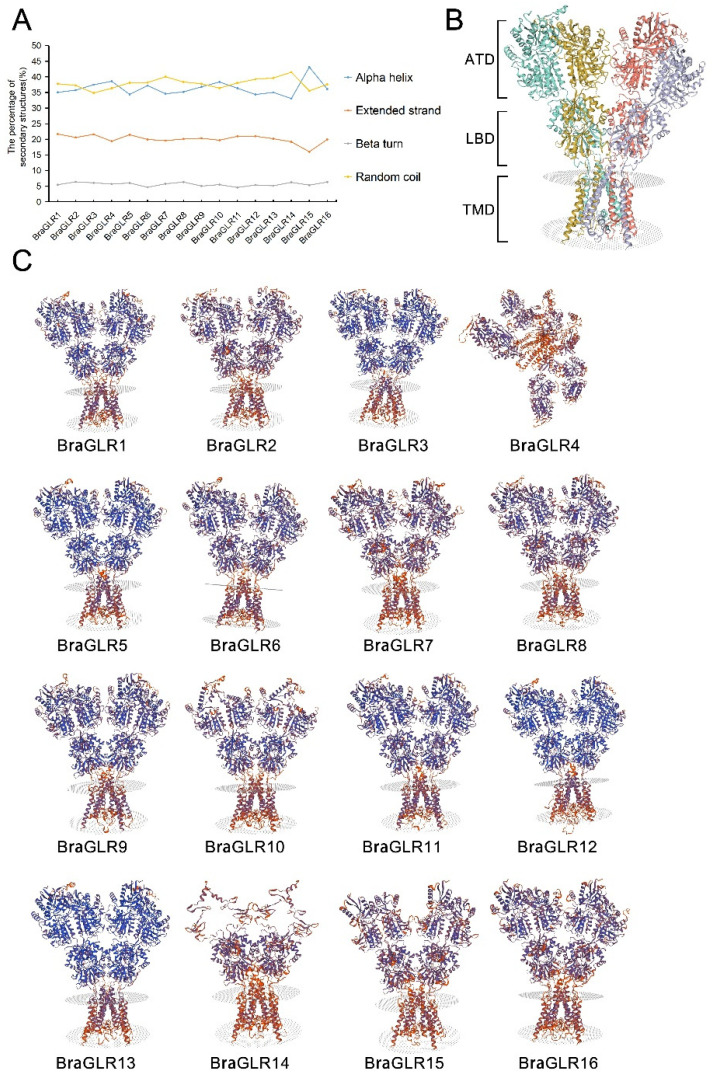
Analysis of BraGLRs’ protein secondary structure and tertiary structure. (**A**) Prediction of secondary structure of GLR proteins in *B. rapa*. (**B**) Predicted tertiary structure of BraGLR1 protein. Four subunits are shown in different colors. The structure of BraGLR1 has a three-layer architecture, which includes the amino-terminal domain (ATD) layer, the ligand-binding domains (LBD) layer, and the transmembrane domain (TMD) layer. (**C**) Prediction of tertiary structure of GLR proteins in *B. rapa*.

The predicted protein secondary structures of BraGLRs were analyzed and all include alpha helix, extended strand, beta turn, and random coil components. The alpha helix and random coil accounted for a larger proportion of the protein secondary structures of BraGLRs, and beta turn accounts for the lowest proportion (Figure 8A).

The tertiary structure of the protein is formed by further coiling and folding on the basis of the secondary structure. Visualizing the tertiary conformation provides strong evidence for understanding the structural properties of its proteins, analyzing the active sites and post-translational modifications to infer the evolutionary relationships among the proteins. Previous studies reveal the tetrameric assembly of AtGLR3.4 subunits into a three-layer domain architecture [38], similar to that of animal ionotropic glutamate receptors. The transmembrane domains (TMDs) form the ion channel, the amino-terminal domains (ATDs) are splayed outward, and the ligand-binding domains (LBDs) are sandwiched in between the ion channel and ATDs (Figure 8B) [39]. In this study, the tertiary structures of BraGLR proteins were predicted using a homology modeling approach (Figure 8C), and the results show that the family contains a large number of random coils and alpha helices, and that most members are structurally similar to AtGLR3.4. This similarity helps provide useful information when studying protein function [40].

### 3.8. Analysis of Protein Interaction Network of BraGLRs

**Figure 9:**
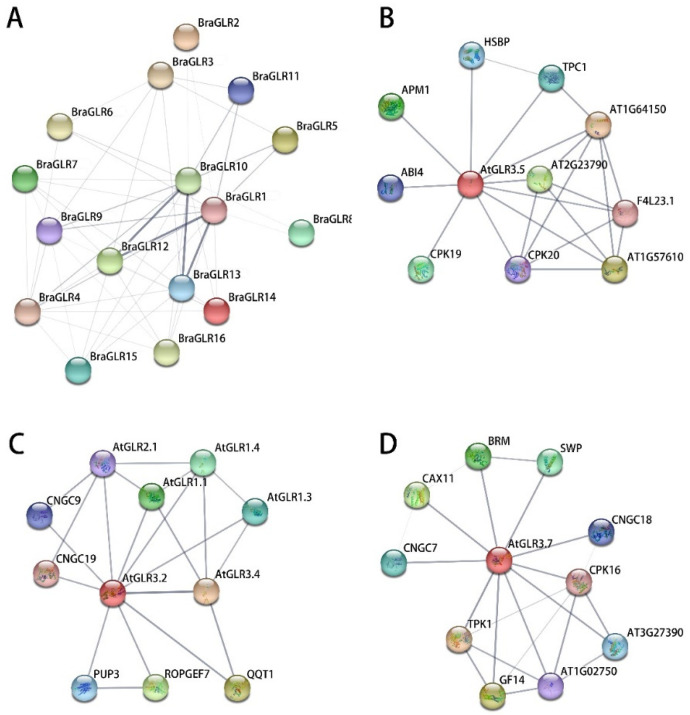
Predictive interaction network of GLRs. (**A**) Predictive analysis of interaction network of GLR proteins in *B. rapa*. (**B**) Predictive analysis of interaction network of BraGLR5 homolog AtGLR3.5. (**C**) Predictive analysis of interaction network of BraGLR1 homolog AtGLR3.2. (**D**) Predictive analysis of interaction network of BraGLR3 homolog AtGLR3.7. Minimum required interaction score of 0.150; default settings were used for the other parameters. Network nodes represent proteins, edges represent protein–protein associations. Minimum required interaction score of 0.150; default settings were used for the other parameters.

Protein is an essential component of all cells and tissues, and is required for all life activities [41]. Few proteins function alone, but instead interact with surrounding proteins to accomplish a range of biological processes, such as DNA transcription and replication, molecular cell signaling, hormone regulation, and metabolism [42]. Proteins can interact in a variety of ways, and any proteins that work together to promote a specific cellular process are considered functionally related [31]. A predicted protein interaction network map of the BraGLRs was constructed based on the resources and algorithms integrated in the STRING database (Figure 9A). Two proteins, BraGLR1 and BraGLR12, have some sequence similarity, so the predicted results show a shared functional association. BraGLR1 and BraGLR10 are both orthologs of AtGLR3.2, and BraGLR9 is orthologous to AtGLR3.3; AtGLR3.2 and AtGLR3.3 mediate long-range signaling in *A. thaliana* [43]. As seen in Figure 8A, both BraGLR1 and BraGLR10 are functionally associated with BraGLR9 to achieve long-range signal transduction. BraGLR12 and BraGLR13 are both orthologs of AtGLR3.4, allowing *A. thaliana* to respond to abiotic stresses such as cold [3]. BraGLR1 and BraGLR10 have a strong functional association with BraGLR12 and BraGLR13, suggesting that the abiotic stress response and long-range signal transduction can function through protein interactions.

BraGLR1 expression was significantly elevated in salt stress. Its homolog in *A. thaliana*, AtGLR3.2, is predicted to interact with cyclic nucleotide-gated channel 19 (AtCNGC19) in *A. thaliana*, AtGLR3.4, and PUP3 (Figure 9B). BraGLR5 was significantly elevated in drought-sensitive plants after drought stress, and the protein interaction network prediction of its homolog AtGLR3.5 revealed that AtGLR3.5 interacted with ABA-related transcription factor ABSCYACID INSENSI-TIVE4 (ABI4), calcium-dependent protein kinase (CPK) family proteins, and calcium uniporter protein AT2G23790, which may interoperate. BraGLR3 expression was elevated after both drought and salt stress, and the protein interaction network prediction of its homolog, AtGLR3.7, revealed that AtGLR3.7 may interact with the drought response family protein, AT1G02750, a CPK family protein, suggesting that BraGLRs may interact with different gene family proteins in response to abiotic stresses.

## 4. Discussion

GLRs activated by glutamate regulate some biological processes in plants by inducing Ca^2+^ inward flow. In plants, both internal plant signals and environmental signals can influence cellular biological processes by affecting [Ca^2+^]_cyt_ [44]. Physiological stimuli such as light, gravity, touch, cold or heat shock, oxidative stress, drought, osmotic shock, hormonal, and salt stress can induce transient increases in [Ca^2+^]_cyt_ [45]. [Ca^2+^]_cyt_ acts as an important second messenger involved in the signal transduction of various environmental stresses. Genes with functions related to resistance or signal transduction in *B. rapa* were identified based on homologous genes in *B. rapa*. To further predict the *GLR* genes in *B. rapa* that may be involved in the regulation of plant growth and development and abiotic stress, we analyzed the predicted protein interaction networks of *B. rapa* and *A. thaliana* and the promoter cis-regulatory elements of *B. rapa* and performed gene expression pattern analysis for abiotic stress and reproductive growth.

According to the transcriptome data analysis, the expression of *BraGLR5* and *BraGLR12* was significantly increased in drought-sensitive plants after being subjected to drought stress. The analysis of promoter elements showed that *BraGLR5* contained drought-inducible and MeJA-responsive elements, and *BraGLR12* contained drought-inducible and ABA-responsive elements. Closure of stomata due to dehydration prevents water loss, and under drought conditions, ABA production and accumulation in plant guard cells contribute to stomatal closure to conserve water. The cellular and molecular mechanisms of ABA-induced stomatal closure have been extensively studied, and ABA is a key hormone in regulating water status and stomatal movement [46]. Drought-tolerant plants respond to drought stress by upregulating genes related to JA and SA metabolism, as well as genes that cause endoplasmic reticulum stress and induce programmed cell death [33]. Plant GLRs also play a crucial role in the salt stress response. The qRT-PCR results show that the relative expression of *BraGLRs* was generally upregulated after the plants were subjected to salt stress. In addition, genes containing a large number of MeJA-responsive elements, such as *BraGLR1*, *BraGLR8*, *BraGLR11*, and *BraGLR16*, were significantly upregulated. In rapeseed (*Brassica napus* L. cv. *Talaye*), the exogenous application of MeJA counteracted the inhibitory effect of NaCl by increasing the soluble sugar content, relative water content, and photosynthetic rate [32]. The expression of genes, such as *BraGLR1*, *BraGLR2*, *BraGLR3*, *BraGLR4*, *BraGLR8*, and *BraGLR10*, containing ABA-responsive elements, was significantly upregulated after salt stress, whereas the *A. thaliana* homolog of *BraGLR3*, *ATGLR3.7*, was shown to be involved in the salt stress and ABA response. Moreover, ABA treatment reduced the expression of AtGLR3.7 [47]. The correlation between GLRs, Ca^2+^, and ABA responses to salt stress during seed germination was studied using the *atglr3.4* mutant, a homolog of *BraGLR12*. The *atglr3.4* mutant was more sensitive to both NaCl and ABA than the wild type. These results suggest that *atglr3.4*-mediated Ca^2+^ inward flow may be involved in the regulation of seed germination under salt stress by regulating Na^+^ accumulation through the salt overly sensitive (SOS) pathway [48].

Protein interactions predicted that AtGLR3.2, a homolog of BraGLR1, which is significantly elevated after salt stress, may interact with AtCNGC19. Previous studies have reported that plant AtCNGCs engage in biotic and abiotic stresses and that AtCNGC19 expression is elevated at increased NaCl concentrations. AtCNGC19 is involved in the tolerance of *A. thaliana* to high salt concentrations [49,50]. Similarly, BraGLR1 may help respond to salt stress by interacting with CNGC family members. The expression of BraGLR5, a homolog of AtGLR3.5, and BraGLR3, a homolog of AtGLR3.7, was elevated after salt and drought stress. The predicted results of the protein interaction network show that, among AtGLR3.5 and ABI4, which may exhibit protein–protein interactions, AtGLR3.5 could increase cytoplasmic Ca^2+^ concentration and repress the expression of ABI4, a key transcription factor involved in the seed ABA response, through calcium ion inward flow, and it plays an important role in regulating Ca^2+^-dependent germination [51]. Both AtGLR3.5 and AtGLR3.7 may interact with genes of the CPK family, and some CDPKs have been shown to be important factors in abiotic stress tolerance, positively or negatively regulating stress tolerance by regulating ABA signaling and reducing reactive oxygen species (ROS) accumulation [52]. Therefore, the phytohormone ABA plays an important role in plant resistance to abiotic stresses. Some CDPKs in *A. thaliana* participate in abiotic and ABA signaling pathways. CPK mutants are involved in guard cell ion channel regulation and ABA-regulated stomatal signaling. The *cpk10* mutant of *A. thaliana* plays a role in ABA- and Ca^2+^-mediated stomatal regulation under drought stress conditions [53]. In *B. rapa*, CDPK, a drought-related transcription factor, was upregulated in drought-tolerant species after 4 h of drought stress [33]. ABA-mediated pathways are thought to play an important role in plant responses to drought and osmotic stress; an ABA-independent pathway has been proposed for the regulation of genes that respond to drought and high salinity but not to cold [54]. AT1G02750, a member of the drought-responsive protein family that interacts with AtGLR3.7, a homolog of BraGLR, is the ABA-independent pathway that binds Zn^2+^ and functions in the drought and salt stress signaling pathway [55]. There is now clear evidence of a metabolically mediated response of the leaf water status in the stomatal response to evaporative demand and soil drought, possibly related to leaf ABA production.

Based on the transcriptome data analysis and qRT-PCR results, *BraGLRs* are involved in the regulation of the growth and development of stamens, and *BraGLR3* may be involved in the growth and development of the pistil. In sexual reproduction, pretreatment of stigma papilla cells with GLR inhibitors reduces self-incompatibility during reproductive growth of Brassicaceae plants, while increasing the [Ca^2+^]_cyt_ of papilla cells before CP decreases self-incompatibility; there is an increased self-incompatibility response in an *AtGLR* mutant with reduced Ca^2+^. Thus, GLRs can mediate Ca^2+^ inward flow, increase [Ca^2+^]_cyt_, and reject self-pollen [13]. Given the relationship between Ca^2+^ and self-incompatibility, we measured the self-incompatibility transcript levels of some members of *BraGLRs* and found that the transcript levels of *BraGLR9* increased significantly in the self-incompatibility (SI) response. The homolog of *BraGLR9* in *A. thaliana* is *AtGLR3.3*, and [Ca^2+^]_cyt_ can be increased due to the direct inward flow of extracellular Ca^2+^ into the cytoplasm via the AtGLR3.3 channel [10]. This analysis reveals that some *BraGLRs* help mediate the Ca^2+^-related self-incompatibility signaling pathway, which could provide directions for exploring plant self-incompatibility signaling.

In summary, we identified 16 *BraGLRs* in the *B. rapa* genome. Based on the comprehensive analysis of sequence features, cis elements, expression profiles of different tissues, abiotic stress tolerance and sexual reproductive processes, and the published data, we further identified *BraGLR1* and *BraGLR9* as playing significant roles in regulating salt stress tolerance and sexual reproduction, respectively.

## Figures and Tables

**Table 1:** Information about GLR gene family members of *B. rapa*.

*BraGLR* Name	*B. rapa* Gene ID	Chromosome	pI	MW (Da)	Protein Length (aa)	Subcellular Location	*A. thaliana* Gene ID	*AtGLR* Name
*BraGLR1*	*Bra011603*	A01:1449556-1453100	8.23	101400.68	913	plas	*AT4G35290*	*AtGLR3.2*
*BraGLR2*	*Bra023313*	A02:2788960-2792786	6.10	97070.42	859	plas	*AT5G11210*	*AtGLR2.5*
*BraGLR3*	*Bra022887*	A03:7572439-7580285	7.59	207961.61	1856	plas	*AT2G32400*	*AtGLR3.7*
*BraGLR4*	*Bra023989*	A03:28396850-28401973	8.18	111000.17	985	plas	*AT4G31710*	*AtGLR2.4*
*BraGLR5*	*Bra005589*	A05:6378019-6381344	7.16	105859.96	945	plas	*AT2G32390*	*AtGLR3.5*
*BraGLR6*	*Bra005591*	A05:6394658-6398235	7.30	102916.70	922	plas	*AT2G32400*	*AtGLR3.7*
*BraGLR7*	*Bra018409*	A05:8057899-8062361	8.45	104777.68	934	nucl, plas	*AT2G29110*	*AtGLR2.8*
*BraGLR8*	*Bra015817*	A07:20705072-20709907	8.64	113543.30	1009	chlo, plas	*AT5G11210*	*AtGLR2.5*
*BraGLR9*	*Bra034931*	A08:5173942-5177131	8.66	103946.12	929	plas	*AT1G42540*	*AtGLR3.3*
*BraGLR10*	*Bra020812*	A08:11978273-11983391	8.40	113307.04	1014	plas	*AT4G35290*	*AtGLR3.2*
*BraGLR11*	*Bra036795*	A09:25762588-25765963	8.65	99347.61	891	plas	*AT3G51480*	*AtGLR3.6*
*BraGLR12*	*Bra032480*	A09:36246731-36249759	7.59	106689.86	961	plas	*AT1G05200*	*AtGLR3.4*
*BraGLR13*	*Bra015398*	A10:2072590-2075666	8.34	106142.33	951	plas	*AT1G05200*	*AtGLR3.4*
*BraGLR14*	*Bra040758*	Scaffold000251:11704-13923	8.77	66681.35	593	plas, E.R.	*AT2G29100*	*AtGLR2.9*
*BraGLR15*	*Bra040760*	Scaffold000251:29107-32937	8.13	93781.66	842	plas	*AT2G29110*	*AtGLR2.8*
*BraGLR16*	*Bra040761*	Scaffold000251:36022-40423	6.85	104916.62	931	cyto, plas	*AT2G29100*	*AtGLR2.9*

## Data Availability

Data are contained within the article/Supplementary Materials.

## Supplementary Materials

The following supporting information can be downloaded at: https://www.mdpi.com/article/10.3390/cells11233729/s1, Table S1: The primers used in the qRT-PCR. Figure S1. Motif sequence.
