# Supplementary material for: Genome-Wide Identification and Expression Analysis of BraGLRs Reveal Their Potential Roles in Abiotic Stress Tolerance and Sexual Reproduction
Source: Cells. 2022 Nov 22;11(23):3729. doi: 10.3390/cells11233729 (PMC9739336; doi:10.3390/cells11233729)
Supplement: Supplementary file 1 [file cells-11-03729-s001.zip › Table S1.pdf]

Table S1. The primers used in the qRT-PCR.

BraGLR **name**	**Forward primer (5´-3´)**	**Reverse primer (5´-3´)**
BraGLR1	GTGTTCATTCACAGCGTTAGAC	CTTGTAAGAGATCTTGCATCGC
BraGLR2	AAACTAATGACCATGCCCACTA	CGATATTGGAGATACGCTAGCT
BraGLR3	TGGCTTTCCGTTACCTTAGATT	TGTGAATTGGTGCATCTTTACG
BraGLR4	TCTGGTGATTTCCGGTTTATCA	CCATATGATTGGTCTGAGTCGA
BraGLR5	CTATGGATCTCTTGCAAGGAGT	TGAAGAAAACTTCAAGAGCACG
BraGLR6	TACATCCATTGCTAATGCGTTG	GAGCCACACATATTCATTGGTC
BraGLR7	GAGCTTAGACCTATGGATCACC	GTTGACTCTATGTTCGAACAGC
BraGLR8	ATCAACGGTCAGTTTTAAAGGC	TCCTAACCTCTTCACTAGTCCA
BraGLR9	TAGTCTCACATCAATCCTGACG	GAAGAGTTCAACATAAGGACGC
BraGLR10	GAGTCTCGTGTAATCATTGTGC	AATCTTCTTGGGAGGTAAAGGG
BraGLR11	ACCTTATGCAGTTCCCTATGAG	CATTGGAACCTAACTTCCGAAC
BraGLR12	AATGAACGATGTAAACGCTGAC	GAATCCACTGCAGTTAGAGTCT
BraGLR13	ATCAAAGACTTGTTGGTTTCCG	CCGTTGATATCCAAACGTAACC
BraGLR14	GTCATTCCTCAATACGGTTTCG	GAAATCAACATACCGTGACCTG
BraGLR15	CTTCAAGACGGCTGGTTTTAAA	TCAAGATAGCCCTTGAGACATC
BraGLR16	TGGAGATGAAGAGCGGATTATC	CGTTGTGGTTGTTACCTTACTG
