# Supplementary figures and images for: Genome-Wide Identification and Expression Analysis of BraGLRs Reveal Their Potential Roles in Abiotic Stress Tolerance and Sexual Reproduction
Source: Cells. 2022 Nov 22;11(23):3729. doi: 10.3390/cells11233729 (PMC9739336; doi:10.3390/cells11233729)

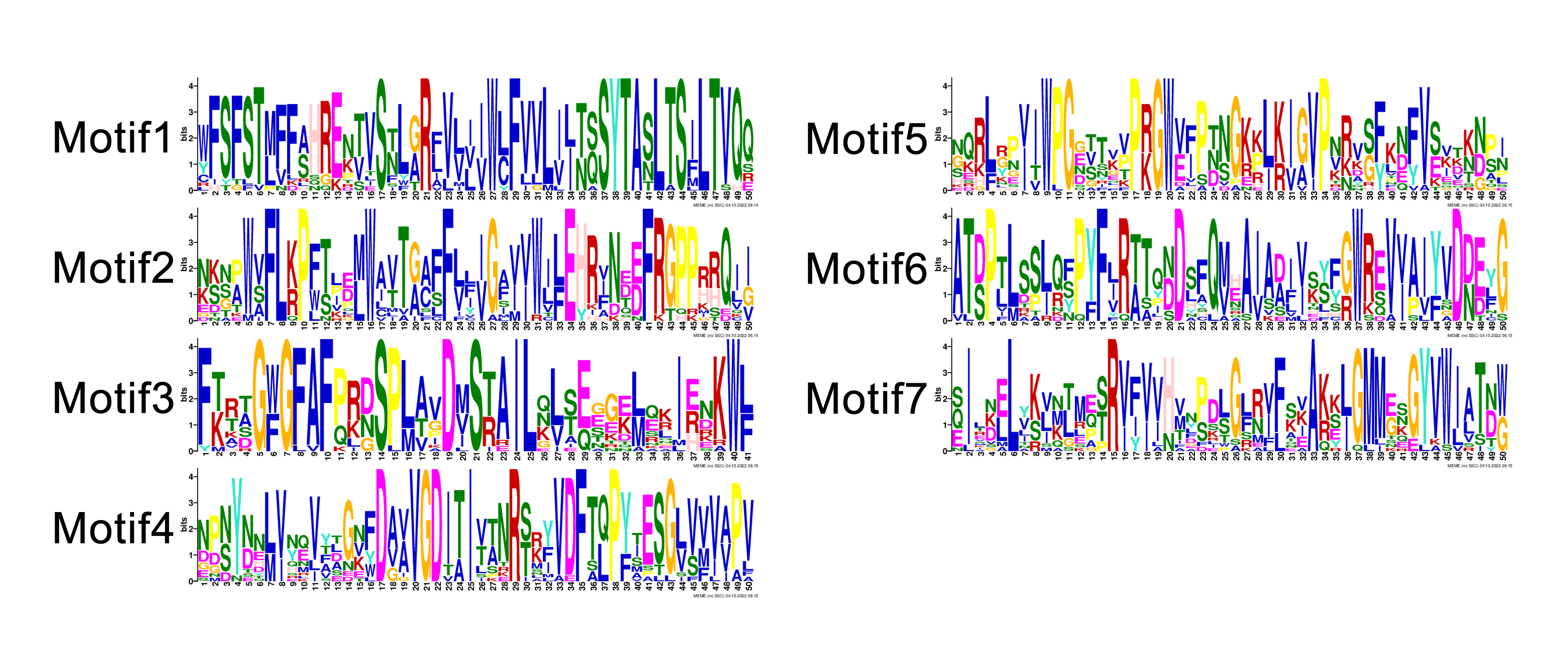

Supplement: Supplementary file 1 [file cells-11-03729-s001.zip › Figure S1.jpg]
